# *Mycobacterium tuberculosis* Multidrug-Resistant Strain M Induces Low IL-8 and Inhibits TNF-*α* Secretion by Bronchial Epithelial Cells Altering Neutrophil Effector Functions

**DOI:** 10.1155/2017/2810606

**Published:** 2017-08-09

**Authors:** Denise Kviatcovsky, Leonardo Rivadeneyra, Luciana Balboa, Noemí Yokobori, Beatriz López, Viviana Ritacco, Mirta Schattner, María del Carmen Sasiain, Silvia de la Barrera

**Affiliations:** ^1^Instituto de Medicina Experimental-CONICET-Academia Nacional de Medicina, Buenos Aires, Argentina; ^2^Instituto Nacional de Enfermedades Infecciosas, ANLIS Carlos G. Malbrán, Buenos Aires, Argentina

## Abstract

M strain, the most prevalent multidrug-resistant strain of *Mycobacterium tuberculosis* (*Mtb*) in Argentina, has mounted mechanisms to evade innate immune response. The role of human bronchial epithelium in *Mtb* infection remains unknown as well as its crosstalk with neutrophils (PMN). In this work, we evaluate whether M and H37Rv strains invade and replicate within bronchial epithelial cell line Calu-6 and how conditioned media (CM) derived from infected cells alter PMN responses. We demonstrated that M infects and survives within Calu-6 without promoting death. CM from M-infected Calu-6 (M-CM) did not attract PMN in correlation with its low IL-8 content compared to H37Rv-CM. Also, PMN activation and ROS production in response to irradiated H37Rv were impaired after treatment with M-CM due to the lack of TNF-*α*. Interestingly, M-CM increased H37Rv replication in PMN which would allow the spreading of mycobacteria upon PMN death and sustain IL-8 release. Thus, our results indicate that even at low invasion/replication rate within Calu-6, M induces the secretion of factors altering the crosstalk between these nonphagocytic cells and PMN, representing an evasion mechanism developed by M strain to persist in the host. These data provide new insights on the role of bronchial epithelium upon M infection.

## 1. Introduction

*Mycobacterium tuberculosis* (*Mtb*), the etiological agent of tuberculosis (TB) is transmitted via inhalation of aerosolized particles. Once inhaled, *Mtb* mainly infects alveolar macrophages as well as nonphagocytic cells lining the alveolar barrier such as M cells, endothelial cells, type II alveolar epithelial cells, and small airway epithelial cells as demonstrated in in vitro infection experiments [1, 2]. Besides, *Mtb* DNA has been isolated from bronchial epithelium of *Mtb* latently infected humans and mice suggesting that mycobacteria can persist intracellularly in nonphagocytic cells from lung tissue even in the absence of histological evidence of immune response [3].

Airway epithelium responds to pathogens by producing antimicrobial molecules, proinflammatory cytokines such as interleukin-1 beta (IL-1*β*), IL-6, tumor necrosis factor alpha (TNF-*α*), and chemokines (IL-8, eotaxin, and macrophage chemoattractant protein-1) which recruit cells belonging to the innate immunity such as macrophages, dendritic cells, and neutrophils (PMN) in order to mount an inflammatory response and control early stages of infection [4, 5].

PMN are the first inflammatory cells that arrive to the site of infection and control microbial infections [6]. Their killing systems are highly efficient and multilayered, including secretion of hydrolytic enzymes contained in granules and antimicrobial peptides, release of neutrophil extracellular traps (NETs), and reactive oxygen species (ROS) production [7]. After phagocytosis, the major neutrophil intracellular killing mechanism is the phagosome-lysosome fusion. In humans, PMN represent the most abundant cell population harboring *Mtb* in bronchoalveolar lavage and sputum samples from patients with active TB [8, 9]. However, the impact of the lung environment on PMN-innate responses during mycobacterial infection remains unknown.

Epidemiological, bacteriological, and genotyping data allowed the identification of the multidrug-resistant (MDR) *Mtb* strain M belonging to the Haarlem family which in the early 1990s was responsible for the MDR outbreak in Buenos Aires area [10]. Thereafter, it disseminated into the community causing 29% of MDR-TB cases during 2003–2008 in Argentina [11]. Increasing evidence has demonstrated that *Mtb* strains with greater dispersal ability are able to manipulate the host immune response for their own benefit. In fact, we have previously demonstrated that in human monocyte-derived macrophages, M grows slowly and promotes low levels of TNF-*α* and IL-10 secretion as well as it is a poor inducer of macrophage cell death suggesting that M strain could remain rather unnoticed by the host macrophage [12]. Besides, M fails to induce ROS and apoptosis in PMN [13], a mechanism that would allow immune evasion and survival of both bacteria and PMN. Thus, as our findings suggests that this strain has mounted several mechanisms to evade conventional innate immune response, the aim of this work was to evaluate whether M strain and H37Rv have the capacity to invade and replicate within nonphagocytic cells, such as the human bronchial epithelial cell line Calu-6, and induce the production of immune mediators. Besides, in order to evaluate the impact of mycobacterial infection on the epithelium-PMN crosstalk, we studied whether conditioned media (CM) derived from M- and H37Rv-infected bronchial epithelial cells are able to attract human PMN to the site of infection and modulate their surface CD11b expression, cell death, and ROS production. We demonstrate that the MDR outbreak strain M is able to infect and survive inside the bronchial epithelial cell line Calu-6 without promoting cell death. Besides, such strain is a low inducer of IL-8, which correlates with an inefficient PMN chemotaxis and inhibits TNF-*α* secretion by the bronchial epithelium. CM from M-infected Calu-6 cells are not able to prime PMN in terms of CD11b expression and ROS production but increases H37Rv replication within bronchial cells. Besides, this CM sustained IL-8 release by H37Rv-infected PMN which could promote the influx of immunocompetent cells to the site of infection.

## 2. Materials and Methods

### 2.1. Donors

Peripheral blood was obtained from BCG-vaccinated healthy donors recruited from Centro Regional de Hemoterapia, Hospital Garrahan, Buenos Aires. All donors gave written informed consent as approved by the research ethics board of the institution. Exclusion criteria included a positive test for human immunodeficiency virus (HIV) and the presence of concurrent infectious diseases or noninfectious conditions like cancer, diabetes, or steroid therapy. The research was carried out in accordance with the Declaration of Helsinki (2013) of the World Medical Association.

### 2.2. *Mycobacterium tuberculosis* Strains

A multidrug-resistant (MDR) strain of *M. tuberculosis* from the collection kept at the Reference Laboratory for Mycobacteria at the INEI-ANLIS “Carlos G. Malbrán” in Buenos Aires and identified on the basis of their spoligotype IS6110 RFLP fingerprint pattern was used: outbreak M strain which belongs to the Haarlem family (isolate 6548). Besides, the reference H37Rv strain (T family) kindly provided by I.N. de Kantor (TB laboratory, INPPAZ PAHO/WHO) was used. Both strains were grown in Middlebrook 7H9 broth base (Sigma Aldrich, St. Louis, MO, USA) with ADC enrichment in agitation for 15–21 days at 37°C in 5% CO_2_ humidified atmosphere until log phase, clumps were disaggregated using glass beads and after 2 h of settling, and the supernatant was harvested, aliquoted, and stored at −80°C until use. In some experiments, H37Rv bacteria were killed by gamma irradiation (2.4 Mrads from a ^137^Cs source) and sonicated and suspended in pyrogen-free phosphate-buffered saline (PBS) 1X at an optical density of 1 OD 600 nm (10^8^ bacilli/ml approximately).

### 2.3. Epithelial Cell Line

The Calu-6 human bronchial epithelial cell line was grown in RPMI 1640 (Gibco Lab., NY, USA) media supplemented with 10% heat-inactivated fetal bovine serum (FBS, Natocor, Argentina), 2 mM L-glutamine, 5.5 mg/ml pyruvate, 100 U/ml penicillin and 100 *μ*g/ml streptomycin (Sigma Aldrich). Cell cultures were maintained till confluence or subconfluence in 75 cm^2^ flasks (Corning CellBIND, NY, USA) at 37°C with a 5% CO_2_-humidified atmosphere. Before using in experiments, cells were detached from the flasks with Trypsin-EDTA 1X (Gibco), washed with RPMI 1640, and seeded at either 24- or 96-well plates (Corning Costar, NY, USA) depending on the test.

### 2.4. Binding and Uptake of *Mtb* Strains by Calu-6 Cells

After 2 h of infection with H37Rv and M strains at a multiplicity of infection (MOI) of 5 bacilli per cell in triplicate, cells were gently washed between 3–5 times with warm PBS 1X to eliminate any remaining bacteria and then added lysis buffer (SDS 0.1%) for 10 min at room temperature followed by neutralization buffer (BSA 20%). An aliquot of this suspension was used to determine the cell-associated colony-forming units (CFU) by plating serial dilutions on 7H11/OADC (Becton Dickinson, MD, USA) agar plates of each *Mtb* strain after 21 days growth.

### 2.5. Intracellular Replication of *Mtb* Strains in Calu-6 Cells

After 2 h of infection with H37Rv and M strains at a MOI of 5 in triplicate, cells were gently washed three times with warm PBS 1X to eliminate any remaining extracellular bacteria and then added warm RPMI 1640 supplemented with 2% FBS to avoid Calu-6 replication during the next 96 h. Once finished with the time of infection, cells were washed 3 times with warm PBS 1X and then added lysis buffer (SDS 0.1%) for 10 min at room temperature and followed by neutralization buffer (BSA 20%). An aliquot of this suspension was used to determine the CFU (denoting combined binding and intracellular replication) by plating serial dilutions on 7H11/OADC (BD Bioscience) agar plates of each *Mtb* strain after 21 days growth.

### 2.6. Conditioned Medium (CM) Derived from Calu-6 Cells Cultures

300.000 adherent cells plated in 24-well plates were infected for 2 h with H37Rv and M strains at a MOI of 5. Afterwards, cells were washed 3 times with warm PBS 1X to eliminate free bacteria and cultured in RPMI 1640 medium with 2% FBS. After incubation at 37°C for 18 h, CM from infected Calu-6 with H37Rv (H37Rv-CM) or M (M-CM) were collected and filtered twice with 0.22 *μ*m membrane pore until use. CM from uninfected Calu-6 cells were used as control (control-CM).

### 2.7. Measurement of Cytokines and Chemokine in CM

The amounts of TNF-*α*, IL-1*β*, IL-10, IL-6, IL-8, GM-CSF, and transforming growth factor beta (TGF-*β*) were determined in control-CM, H37Rv-CM, and M-CM by ELISA, according to manufacturer's instructions (TNF-*α*, IL-1*β*, IL-8, and TGF-*β*, eBioscience, San Diego, CA, USA; IL-10, IL-6, and GM-CSF, ELISA MAX™ Sets BioLegend, San Diego, CA, USA).

### 2.8. Depletion of TNF-*α* in CM

The depletion was followed as described previously [14]. H37Rv-CM was incubated with neutralizing TNF-*α* antibody (10 *μ*g/ml, clone TNF 104C, mouse IgG2a, BioLegend) for 1 h at 4°C and then added to 100 *μ*l/ml of previously washed Protein G sepharose beads (Amersham Pharmacia Biotech AB, Sweden). Upon 1 h incubation at 4°C, H37Rv-CM was centrifuged (12,000*g*) to remove antibody-bead complexes before use. The depletion was confirmed by ELISA.

### 2.9. Isolation of Human PMN

PMN were isolated from peripheral blood by Ficoll Hypaque (Sigma Aldrich) gradient centrifugation followed by dextran 6% (Sigma Aldrich) sedimentation as previously described [15]. Cell suspensions contained 96% PMN as determined by May Grunwald-Giemsa staining in cytopreps and the levels of monocyte contamination were always <0.2%, as evaluated by CD14 staining and flow cytometry (FACS) analysis. The cells were suspended in RPMI 1640 medium supplemented with 2% FBS at a concentration of 1 × 10^7^ cells/ml.

### 2.10. Determination of Adherence/Entrance and Replication of H37Rv in PMN

Infection of PMN with H37Rv strain was performed according to Morris et al. [16] with minor modifications. Briefly, purified PMN were suspended in RPMI 1640 medium supplemented with 10% FBS. 1 × 10^5^ cells were added to each well of 96-well tissue culture plates (Corning Costar) previously coated with poly-L-lysine (Sigma Aldrich) and incubated for 18 h at 37°C, 5% CO_2_. Then, cells were gently washed once with warm PBS 1X to remove nonadherent cells. Attached PMN were treated for 1.5 h with the CM and infected with H37Rv at a MOI of 3 for 1 h to allow phagocytosis and uptake. Infected PMN were washed 3 times with warm PBS 1X to eliminate nonphagocytosed bacteria and treated with lysis buffer (SDS 0.1%) for 10 min at room temperature followed by addition of neutralization buffer (BSA 20%). To evaluate the replication of H37Rv within PMN, infected PMN were cultured for further 18 h in RPMI 1640 medium supplemented with 5% FBS and gentamicin (50 *μ*g/ml, Sigma Aldrich) which allow the clearance of the remaining extracellular bacteria. Then, cells were lysed as previously described. In both cases, an aliquot of this suspension was used to determine the CFU by plating serial dilutions on 7H11/OADC (BD Bioscience) agar plates after 21 days growth.

### 2.11. PMN Chemotaxis Assay

Fresh PMN were suspended at a density of 1 × 10^5^ PMN/ml in RPMI 1640 plus 2% FBS, and chemotaxis was quantified using a modification of the Boyden chamber technique [17]. PMN suspensions were placed in the upper wells of a 18-well microchemotaxis chamber (Neuro Probe Inc., Bethesda, Maryland, USA) separated by a polyvinylpyrrolidone- (PVP-) free polycarbonate filter (3 *μ*m pore size; Poretics Products, Livermore, California, USA). The lower wells contained the CM or N-Formylmethionine-leucyl-phenylalanine (fMLP) 10^−7^ M as positive control. The chamber was incubated for 1.5 h at 37°C in a 5% CO_2_-humidified atmosphere. Following incubation, the chamber was disassembled and the membrane filter was removed, fixed, and stained with Staining 15 (Biopur Diagnostics, Argentina). The number of PMN on the undersurface of the chamber filter was counted in five random high-power fields (HPF) at a magnification of ×400 for each of triplicate filters.

### 2.12. Culture of PMN with Calu-6 Cell-Derived CM

1 × 10^5^ PMN were treated for 1.5 h with control-CM or H37Rv-CM or M-CM, and then they were stimulated or not for further 3 h with gamma-irradiated H37Rv (5 i-H37Rv : 1PMN ratio). PMN cultured in RPMI 1640 plus 2% FBS (N-T) were also incubated with PMA (50 nM for 30 min, Sigma Aldrich) or TNF-*α* (20 ng/ml, BioLegend) as positive controls. Afterwards, harvested cells were tested for surface CD11b expression and ROS production. In parallel experiments, PMN previously stimulated or not with CM were infected or not with live H37Rv (MOI of 3) and cultured for further 1 or 18 h. Then, PMN were collected and tested for bacilli uptake and intracellular replication rate. Supernatants were tested for LDH and IL-8 release.

### 2.13. PMN Phagocytosis

The ability of PMN, previously exposed or not to control-CM or *Mtb*-CM, to capture *Mtb* was determined by measuring uptake of FITC-labeled i-H37Rv as described previously [18]. Briefly, 10^8^ bacteria/ml were incubated with FITC (0.5 mg/ml in PBS, Sigma Aldrich) at room temperature for 1 h. Then, stained bacilli were washed four times to remove unbound FITC and suspended in RPMI 1640 containing 10% FBS. PMN (1 × 10^5^) incubated with control-CM, H37Rv-CM, and M-CM for 1.5 h were cultured with FITC-i-H37Rv (5 i-H37Rv : 1 PMN ratio) for 1 h at 37°C. After that, cells were extensively washed to remove extracellular bacteria and the percentage of PMN that phagocytosed FITC-i-H37Rv was measured by FACS. Control experiments were performed using PMN pretreated for 30 min before adding the FITC-labeled i-H37Rv with cytochalasin B (5 *μ*g/ml, Sigma Aldrich), which inhibits network formation by actin filaments.

### 2.14. Oxidative Burst Assay

Intracellular ROS production was measured by Dihydrorhodamine 123 assay (DHR, Sigma Aldrich) as previously described [19]. Briefly, 1 × 10^5^ PMN were stimulated for 1.5 h with CM from *Mtb*-infected or *Mtb*-noninfected Calu-6 cells. Thereafter, cells were incubated with or without a suboptimal concentration of 5 nM PMA at 37°C for 30 min or i-H37Rv (5 : 1 *Mtb* : PMN ratio) for 3 h to induce oxidative stress and then labeled with DHR (5 *μ*M, Sigma Aldrich) for 30 min. Afterwards, culture was stopped by incubating the samples at 4°C and analyzed immediately in a FACScan flow cytometer (Becton Dickinson) by determining DHR 123 conversion to rhodamine 123 (DHR MFI). Fluorescence was measured on FL-1 in 10.000 events and data were analyzed with FCS Express software (De Novo Software, USA).

### 2.15. Calu-6 and PMN Surface Phenotypic Analysis by Flow Cytometry

- Calu-6 cells infected or not were detached from the plates and treated with 4% paraformaldehyde (PFA, Sigma Aldrich) previously to staining. Then, were washed with warm PBS 1X and stained for 30 min at 4°C with PE-anti-human CD54 (eBioscience), PE-anti-human mannose receptor, PE/Cy5-anti-CD11b, FITC-anti-TLR2 (BioLegend), and their corresponding isotypes.
- PMN treated or not with CM and then infected with H37Rv (MOI of 3) were treated with 4% PFA and then stained with PE/Cy5-anti-CD11b, PE-anti-TLR2 (BioLegend), and their corresponding isotypes.

Stained PMN and Calu-6 cells were washed, fixed with 0.5% PFA, and suspended in IsoFlow™ (BD Bioscience) for acquisition in a FACScan flow cytometer (Becton Dickinson). In both cases, 10.000 events were acquired in Calu-6 and PMN gates set according to forward- and side-scatter properties. Data were analyzed with FCS Express software (De Novo Software, USA). Results are expressed as mean fluorescence intensity (MFI) or relative MFI (MFI from *Mtb*-treated cells/MFI from untreated cells).

### 2.16. Detection of *Mtb*-Induced Apoptosis/Necrosis in Calu-6 Cells and PMN by Flow Cytometry

Calu-6 cells infected or not with the strains (MOI of 5) and PMN treated or not with CM and then infected with H37Rv (MOI of 3) were cultured for further 18 h, detached from the 96-well tissue culture plates, and washed to obtain the pellet. Then Calu-6 and PMN cellular pellets were stained with a fixable viability dye FVD eFluor 780 (eBioscience) and vortexed immediately. After 30 min of incubation at −4°C and protected from light, cells were washed with Binding Buffer 1X (Sigma) and stained with Annexin V kit (Sigma Aldrich) according to the manufacturer instructions. After the time of incubation with Annexin V, cells were washed and fixed with PFA 1% for 20 min and washed with Binding Buffer 1X (Sigma Aldrich). Finally, cells were suspended in flow cytometry buffer and acquired at the cytometer. Results are expressed as percentage of positive or negative cells for Annexin V and FVD eFluor 780.

### 2.17. LDH Secretion Test in Calu-6 Cells and PMN

To analyze the effect of *Mtb* infection on Calu-6 cell permeability, the release of lactate dehydrogenase (LDH) was determined in CM from 18 h-cultured uninfected (low controls) and *Mtb*-infected Calu-6 cells (2 h infection at a MOI of 5) (experimental values). Besides, the release of LDH was measured in supernatants derived from PMN previously incubated with the CM for 1.5 h and infected or not with H37Rv (MOI of 3) for 1 h, washed, and finally cultured for further 18 h (experimental values). Supernatant from 18 h-cultured PMN N-T and uninfected cells was employed as low control. Maximum LDH release (high control) was determined in supernatants from Calu-6 cells and PMN treated with Triton X100 for 15 min at 37°C. In all cases, culture supernatants were collected and filtered twice. LDH levels were measured with a kinetic UV method kit (Roche Diagnostics GmbH, Germany). The percentage of specific LDH release was determined as %LDH release = [(OD_490_ experimental values − OD_490_ low control)/(OD_490_ high control − OD_490_ low control)] × 100.

### 2.18. Measurement of NO in Supernatants from PMN

NO production by PMN treated or not with CM and infected with H37Rv at a MOI of 3 for 1 h and further 18 h of culture was performed using an indirect method based on measurement of nitrite concentration in culture supernatants according to Griess reaction [20]. Nitrite concentrations were determined by spectrophotometric analysis at 540 nm with extrapolation from a standard curve prepared in parallel. Nitric oxide (NO) products were expressed as *μ*M in the culture media supernatants.

### 2.19. Measurement of the IL-8 Amount in Supernatants from PMN

PMN untreated or incubated with the CM were infected or not with H37Rv (MOI of 3) and then cultured for further 18 h. Then, supernatants were collected and filtered twice with a 0.22 *μ*m pore membrane. IL-8 was determined by ELISA (BioLegend) according to manufacturer's instructions.

### 2.20. Statistical Analyses

Results were expressed as mean and SEM. Data analysis was performed using the one-way ANOVA nonparametric Friedman test followed by Dunn's multiple comparison test. The significance level adopted was *p* < 0.05. All the statistical analyses were performed with the GraphPad software (San Diego, CA).

## 3. Results

### 3.1. M Strain Is Less Effective to Invade but Survives within Calu-6 Cells

**Figure 1:**
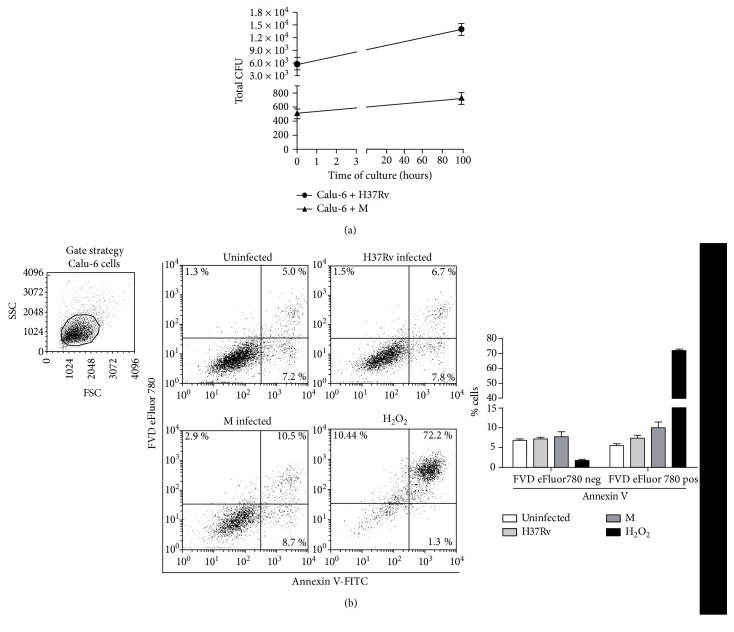
*Mycobacterium tuberculosis* strains invade and replicate within Calu-6 cells. Calu-6 cell monolayers were infected with H37Rv (●) or M (▲) strains at a MOI of 5 for 2 h and (a) either harvested and lysed to determine bacterial uptake or cultured for further 96 h to allow intracellular replication. Cells were lysed at each time point and serial dilutions were plated to determine CFU. Total CFU from Calu-6 cells cultured at 0 h or 96 h upon infection; mean ± SEM are shown (*n* = 6) or (b) allow intracellular replication for further 18 h to determine cell death by measuring Annexin V and FVD eFluor 780 expression by flow cytometry. Results are expressed as percentage of early apoptotic (Annexin Vpos/FVD eFluor780neg), late apoptotic (Annexin Vpos/FVD eFluor 780pos), or necrotic cells (Annexin Vneg/FVD eFluor 780pos). H_2_O_2_ was used as apoptosis positive control. Gate strategy for Calu-6 cells is shown.

We first evaluated whether H37Rv and M strains were able to bind and/or internalize into nonphagocytic cells such as the human bronchial epithelial cell line Calu-6. As observed in Figure 1(a), both strains were able to bind to and/or invade Calu-6 but M strain was in a lower rate than H37Rv as detected in CFU assays. Interestingly, although both strains survive within bronchial cells, bacteria recovered from 96 h-cultured M-infected cells were low, unlike H37Rv. These results suggest that M strain is less efficient to invade and replicate but is able to survive within bronchial epithelium.

It has been demonstrated that H37Rv induces necrosis of infected alveolar epithelial cells [21], so we decided to evaluate whether H37Rv and M strains differed in their ability to cause apoptosis and/or necrosis on Calu-6 cells. Both strains induced a slight increase in the percentage of early (Annexin Vpos /FVD eFluor 780neg) and late apoptotic (Annexin Vpos /FVD eFluor 780pos) cells but did not induce necrosis (Annexin Vneg /FVD eFluor 780pos) at a MOI of 5 (Figure 1(b)) or even at a MOI of 50 (data not shown).

### 3.2. M Strain Diminishes TNF-*α* and GM-CSF Release and Induces Low IL-8 Secretion by Calu-6 without Altering Surface Receptor Expression or Cell Survival

Human bronchial epithelial cells are able to recognize Gram-positive and Gram-negative bacteria through different pattern recognition receptors (PRRs) [22], so we first evaluated the expression of *Mtb* recognizing PRRs such as TLR-2, MR, CD11b, and CD54 in 18 h-cultured uninfected and *Mtb*-infected Calu-6. Low expression of these markers was observed on either infected or uninfected cells (Supplementary Figure 1 available online at https://doi.org/10.1155/2017/2810606).

**Figure 2:**
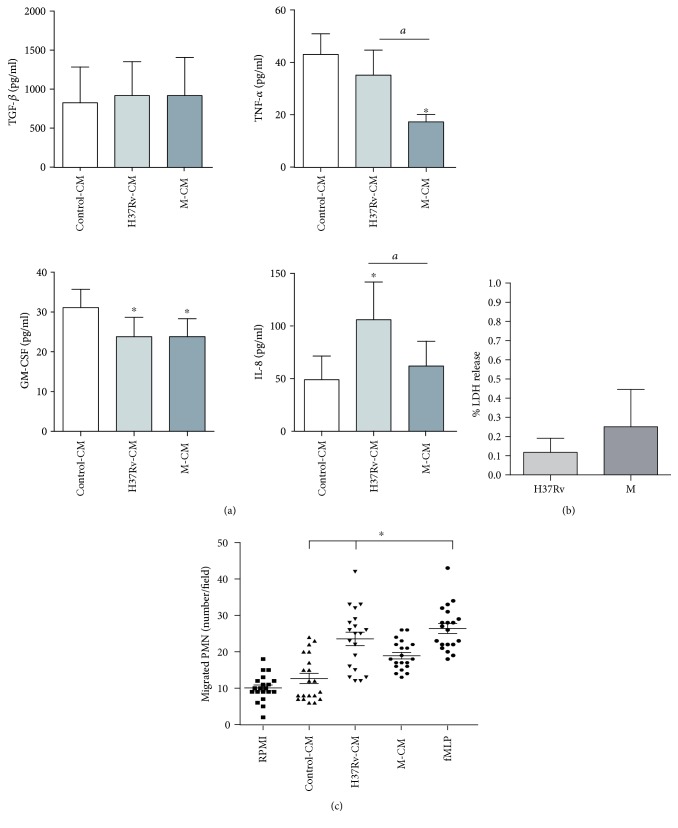
Characterization of conditioned media from infected Calu-6. Conditioned media (CM) from 18 h cultured uninfected (control-CM) or H37Rv- or M-infected Calu-6 cells (H37Rv-CM and M-CM) were tested for (a) TGF-*β*, TNF-*α*, GM-CSF, and IL-8 by ELISA. Results are expressed as pg/ml and mean ± SEM. Statistical differences: ^∗^*p* < 0.05 for H37Rv-CM or M-CM versus control-CM, ^*a*^*p* < 0.05 for M-CM versus H37Rv-CM. (b) LDH release by employing a kinetic UV assay. Triplicates for each condition were performed. Percentage of LDH release was calculated as described in Materials and Methods. Results expressed as mean ± SEM (*n* = 8). (c) Ability to attract resting PMN by employing a PMN chemotaxis assay and fMLP (10^−7^ M) as positive control. Migrated PMN were counted by light microscopy. Five random fields per condition (each condition by triplicated) were analyzed, and results were expressed as number of migrated PMN/field (mean and SEM). Statistical differences: ^∗^*p* < 0.05 for H37Rv-CM or M-CM versus control-CM.

Thereafter, the secretion of the proinflammatory IL-1*β*, IL-6, GM-CSF, and TNF-*α* and anti-inflammatory cytokines IL-10 and TGF-*β* as well as the chemokine IL-8 was measured in CM from noninfected (control) or H37Rv- and M-infected Calu-6. In our system, IL-1-*β*, IL-6, and IL-10 were not detectable (data not shown). In contrast, TGF-*β* secretion was detected in control-CM and *Mtb* infection did not modify its amounts (Figure 2(a)). Interestingly, the high level of TNF-*α* in control-CM was not increased by H37Rv infection, while a significant reduction in M-CM was detected. Additionally, CM derived from infected Calu-6 showed an inhibition of GM-CSF secretion when compared to noninfected cells, but no differences were observed among strains. Furthermore, increased IL-8 levels were detected in H37Rv-CM unlike M-CM. Thus, M-CM induced lesser levels of proinflammatory mediators than H37Rv-CM in the bronchial epithelium.

In order to determine if the low cytokine production induced by M is due to a specific immunosuppressive mechanism or merely to the fact that M is tenfold lesser infective than H37Rv, TNF-*α*, GM-CSF, and IL-8 amounts were determined in CM derived from Calu-6 cells infected at a MOI of 50. As observed in Supplementary Figure 2, TNF-*α* and IL-8 release was dependent on the number of infecting bacilli within epithelial cells and due to the poor infecting capacity of M, cells infected with this strain at a MOI of 50 released similar levels of TNF-*α* and IL-8 than those infected with H37Rv at a MOI of 5. Regarding GM-CSF, and in contrast to H37Rv, M induced low levels of this cytokine despite the MOI employed suggesting that M could be specifically inhibiting GM-CSF production.

We also measured the release of lactate dehydrogenase (LDH), a soluble factor indicative of cell permeability in CM from 18 h-cultured infected Calu-6. As shown in Figure 2(b), and in concordance to apoptosis data, the release of LDH by *Mtb* strains was negligible.

### 3.3. CM from M-Infected Calu-6 Show Low PMN Chemoattractant Activity

As human-circulating PMN enter the site of infection by mainly migrating along a gradient of IL-8 [23] and as different amounts of IL-8 were found in CM induced by either M or H37Rv strains (MOI of 5), we evaluated the PMN chemoattractant capacity of CM derived from *Mtb*-infected Calu-6. As shown in Figure 2(c), H37Rv-CM and fMLP (positive control) induced higher numbers of migrated PMN than control-CM and M-CM, which may be partially ascribed to their IL-8 levels.

### 3.4. CM Alter PMN Activation by i-H37Rv

**Figure 3:**
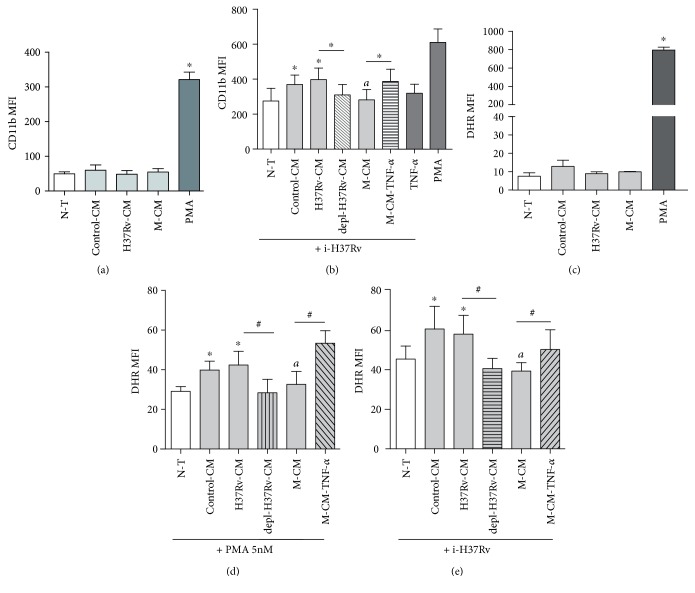
CM alter PMN activation and ROS production by irradiated H37Rv (i-H37Rv). Freshly, PMN (*n* = 7) were incubated for 1.5 h with (a) RPMI 1640 plus (N-T) or with conditioned media (CM) from Calu-6 cells infected with H37Rv or M (H37Rv-CM and M-CM) or noninfected (control-CM) or (b) additionally with H37Rv-CM-depleted TNF-*α* (depl-H37Rv-CM), M-CM plus TNF-*α* (M-CM-TNF-*α*), or TNF-*α* (20 ng/ml). Thereafter, PMN were stimulated for further 3 h with or without i-H37Rv or PMA (5 nM) as a second stimulus. PMA (50 nM) was employed as positive control for the last 30 min. PMN were tested for CD11b expression (a) and ROS production (b) by labeling with DHR for 30 min (c, d, and e) by FACS. Results are expressed as median fluorescence intensity (MFI) ± SEM. (a) Statistical differences: ^∗^*p* < 0.05 for PMA versus nontreated (N-T) or CM-treated PMN. (b) Statistical differences: ^∗^*p* < 0.05 for CM-treated PMN versus N-T PMN; ^#^*p* < 0.05 for PMN treated with depl-H37Rv-CM versus H37Rv-CM or PMN treated with M-CM plus TNF-*α* versus M-CM; ^*a*^*p* < 0.05 for M-CM versus control-CM or H37Rv-CM. (c) Statistical differences: ^∗^*p* < 0.05 for PMA versus N-T or CM-treated PMN. (d and e) Statistical differences for PMA and i-H37Rv, respectively: ^∗^*p* < 0.05 for treated PMN versus nontreated PMN; ^#^*p* < 0.05 for PMN treated with depleted-H37Rv-CM versus H37Rv-CM or PMN treated with M-CM plus TNF-*α* versus M-CM; ^*a*^*p* < 0.05 for M-CM versus control-CM or H37Rv-CM.

Once arrived to the site of infection, PMN encounter an inflammatory environment enriched in host-derived cytokines and pathogen-derived chemoattractants that can modulate their responsiveness to subsequent pathogen stimuli. So, we first assessed if CM exerted an effect on CD11b expression which is upregulated upon PMN activation [24]. As we can observe in Figure 3(a), the treatment of PMN with CM derived from cells infected at a MOI of 5 did not modify the expression of CD11b, while it was significantly enhanced after PMA stimulation.

To determine if CM are able to prime PMN, cells were incubated with the CM and then exposed to i-H37Rv as second stimuli. PMN treated with control-CM or H37Rv-CM and then exposed to i-H37Rv showed a higher CD11b expression than N-T PMN, while M-CM did not alter i-H37Rv-induced CD11b upregulation (Figure 3(b)). Given that TNF-*α* is known to prime PMN functional responses [25] and CM present different amounts of this cytokine, we first evaluated whether CD11b enhancement could be due to TNF-*α* secreted by Calu-6. For this purpose, H37Rv-CM depleted from or M-CM supplemented with TNF-*α* were used to stimulate PMN and then exposed to i-H37Rv. The depletion of TNF-*α* from H37Rv-CM resulted in a reduction of CD11b expression when PMN were exposed to i-H37Rv compared to that from nondepleted CM (Figure 3(b)). In opposition, M-CM plus TNF-*α* increased CD11b expression of PMN in comparison to M-CM alone. These results suggest that TNF-*α* released to the extracellular environment is involved in the activation of PMN at least in terms of CD11b expression. Although, we cannot exclude that other soluble factors are implicated.

### 3.5. CM from M-Infected Bronchial Cells Do Not Enhance i-H37Rv-Induced ROS Production by PMN

As PMN are highly effective to generate ROS upon activation [26], we further evaluated whether CM altered intracellular ROS production by PMN. For this purpose, PMN were treated with the CM and then stimulated or not with suboptimal doses of PMA or i-H37Rv and ROS production was evaluated by DHR assay. Exposure of PMN to CM resulted in negligible ROS production although optimal doses of PMA significantly did (Figure 3(c)). However, PMN incubated with control-CM or H37Rv-CM enhanced ROS production in response to subsequent challenge with suboptimal PMA concentration (Figure 3(d)) or i-H37Rv (Figure 3(e)) compared to N-T PMN. In contrast, M-CM was not able to enhance ROS production in response to PMA or i-H37Rv. To determine whether TNF-*α* was involved the CM priming effect in ROS production, PMN were incubated with TNF-*α*-depleted H37Rv-CM or M-CM plus TNF-*α* and then stimulated with i-H37Rv. The depletion of TNF-*α* treated with H37Rv-CM reduced ROS production from PMN than that treated with H37Rv-CM, while higher ROS was observed by addition of TNF-*α* to M-CM compared to M-CM alone (Figure 3(e). These results suggest that TNF-*α* released by infected bronchial epithelial cells would indirectly manipulate ROS production by PMN in response to specific and unspecific stimuli. Indeed, M strain may be able to impair PMN effector functions through a bystander effect in which infected bronchial epithelial cells secrete poor levels of TNF-*α*.

### 3.6. CM from *Mtb*-Infected Bronchial Epithelial Cells Do Not Modify i-H37Rv Phagocytosis by PMN

**Figure 4:**
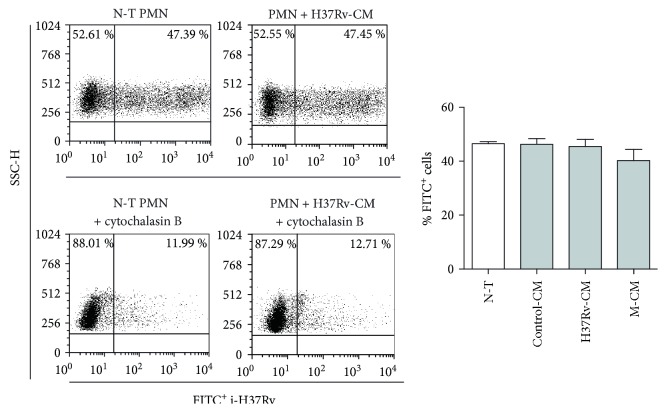
Impact of CM on i-H37Rv phagocytosis by PMN. PMN (*n* = 6) cultured for 1.5 h alone (N-T) or with control-CM, H37Rv-CM, or M-CM in the presence or not of cytochalasin B were incubated for 1 h with FITC-labeled i-H37Rv. Then, the percentage of cells that phagocytosed FITC-i-H37Rv was determined by FACS. Median ± SEM of one representative experiment is shown.

ROS production is associated with the efficiency of the pathogen to enter to PMN [13, 27]. For this reason, we wondered whether CM altered i-H37Rv phagocytosis by PMN. So, PMN incubated with CM were cultured with FITC-labelled i-H37Rv, and the percentage of cells that phagocytosed the bacteria (FITC^+^ cells) was measured by FACS. As shown in Figure 4(a), the treatment of PMN with CM did not modify the percentage of FITC^+^ cells while phagocytosis was indeed abolished by cytochalasin B, suggesting that soluble mediators released by the infection would not influence the phagocytosis of i-H37Rv by PMN.

### 3.7. M-CM Enhances H37Rv Replication within PMN

**Figure 5:**
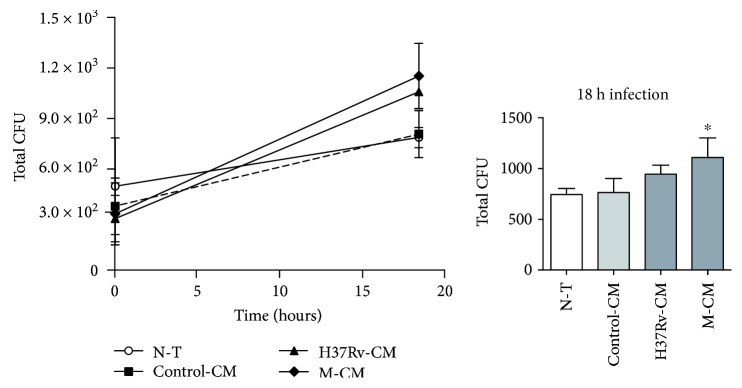
Impact of CM on uptake/replication of H37Rv within PMN. PMN (*n* = 7) cultured for 1.5 h alone (N-T) or with control-CM, H37Rv-CM, or M-CM were infected or not with H37Rv at MOI of 3 for 1 h. Then noninfected or H37Rv-infected PMN were collected (0 h) or cultured for further 18 h. Numbers of CFU recovered from 0 h (uptake) or 18 h upon infection were determined as described in Materials and Methods. Median ± SEM is shown. Statistical differences: ^∗^*p* < 0.05 for PMN preincubated with M-CM versus NT-PMN.

We also wondered whether CM modified the rate of PMN invasion and replication by live H37Rv. To do so, PMN pretreated with the CM were infected with H37Rv and CFU were determined at 0 h (mycobacterial uptake) or 18 h (mycobacterial replication and survival) upon infection. As shown in Figure 5(a), no differences in mycobacterial uptake were observed between PMN treated with the CM or untreated, being these results were in agreement with phagocytosis data. Indeed, PMN exposed to M-CM were more permissive to H37Rv replication than the N-T ones.

### 3.8. Impact of CM on PMN Survival, NO Production, and IL-8 Release upon *Mtb* Infection

**Figure 6:**
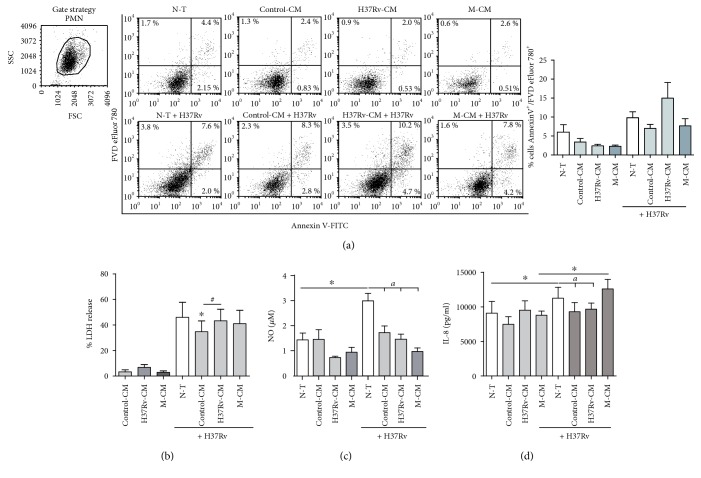
Impact of CM on PMN cell death, NO production, and IL-8 secretion. (a) PMN treated or not with CM and infected with H37Rv (MOI = 3) tested the expression of Annexin V and FVD eFluor 780 by flow cytometry. The percentage of early apoptotic (Annexin V pos/FVD eFluor 780neg), late apoptotic (Annexin Vpos/FVD eFluor 780pos), and necrotic (Annexin Vneg/FVD eFluor 780pos) cells was determined. Dot plots from a representative experiment out of 5 done and a bar graph showing the percentage of late apoptotic cells (mean ± SEM) are displayed (b–d). Supernatants derived from H37Rv-infected PMN were recovered at 18 h postinfection and tested for (b) LDH release, expressed as percentage (median ± SEM). Statistical differences: ^∗^*p* < 0.05 for control-CM-treated PMN versus N-T PMN; ^#^*p* < 0.05 H37Rv-CM versus control-CM-treated PMN (*n* = 7), (c) reactive nitrogen species (NO), expressed as *μ*M (median ± SEM) (*n* = 5) and (d) IL-8 secretion expressed as pg/ml (mean ± SEM). Statistical differences: ^∗^*p* < 0.05 for H37Rv-infected versus noninfected PMN; ^*a*^*p* < 0.05 for CM-treated PMN versus N-T PMN (*n* = 7).

As PMN quickly succumb to cell death upon H37Rv infection [28], we wondered whether CM from bronchial epithelial cells could rescue PMN from cell death. For this purpose, PMN were stimulated or not with CM and infected with H37Rv at a MOI of 3 and then cultured for further 18 h. As shown in Figure 6(a), infected PMN showed higher percentage of cells undergoing late apoptosis than that of uninfected cells while no differences were observed in necrosis and early apoptosis. Pretreatment of PMN with control-CM and M-CM diminished the percentage of late apoptotic PMN when compared to nontreated cells while H37Rv-CM did not. In the same context, infected PMN showed higher percentage of LDH release than noninfected cells and pretreatment of PMN with control-CM diminished LDH secretion while H37Rv-CM and M-CM did not (Figure 6(b)). This fact could be connected with the amounts of GM-CSF from CM.

It has been proposed that reactive nitrogen intermediates have a bacteriostatic effect on *Mtb* in cell-free systems [29], so we wondered whether PMN pretreated with CM differed in their ability to produce NO. We observed that H37Rv infection enhanced NO production in nonexposed PMN while pretreatment with CM diminished its production upon infection (Figure 6(c)).

We further determine if CM modulate IL-8 secretion by PMN infected or not with H37Rv upon an 18 h culture. As observed in Figure 6(d), no differences in IL-8 production were observed between PMN preincubated with the CM and N-T PMN. An increase in IL-8 production was detected in N-T PMN upon H37Rv infection when compared with that in the uninfected ones. While PMN exposed to M-CM displayed similar IL-8 amounts when compared to N-T PMN, preincubation with control-CM or H37Rv-CM showed lower IL-8 secretion upon H37Rv infection.

## 4. Discussion

In vitro and in vivo evidences suggest that bronchial epithelial cells are highly likely to encounter *Mtb* before its arrival to the alveolar space [3, 30]. Therefore, bronchial cells may contribute to the initial clearance of the pathogen by secreting antimicrobial peptides and influence the early immune response by releasing cytokines and chemokines which sustain the influx of cells from the innate and adaptive immune system to the site of infection and their consequent activation [31]. In this study, we report that the MDR *Mtb* strain M is able to infect and survive within cells from the bronchial epithelial cell line Calu-6. In addition, CM from M-infected Calu-6 showed poor chemoattractant activity of PMN due to the low levels of secreted IL-8 which could diminish the recruitment of PMN to the infected lung compartment and, furthermore, inhibit TNF-*α* secretion by Calu-6 altering therefore the bystander activation of PMN.

It has been established that bronchial cells are less permissive to invasion than alveolar cells [30]. Accordingly, in this study, we observed that Calu-6 cells are less permissive to *Mtb* invasion since from the initial inoculum, 21% of H37Rv and 8% of M were uptaken by these cells and, additionally, denote the importance of the mycobacteria genotype in the invasion of the bronchial epithelium. In this line, it has been proposed that interaction with airway epithelium greatly depends on the virulence phenotype of *Mtb*. In this way, F15/LAM4/KZN isolated from KwaZulu-Natal and Beijing strains with a high rate of spreading into the community displays greater adhesion to and infection of human alveolar and bronchial epithelial cells than those from unique fingerprinting or H37Rv strains [32]. In contrast with these results, we observed that M strain, that is highly successful to spread into the community [33], displayed low ability to replicate within Calu-6, suggesting that this low-replicating strain would have developed evasion mechanisms to survive in a nonconventional niche. On the other hand, slight Calu-6 detachment of the monolayers was observed with either H37Rv or M strain (data not shown) that could not be ascribed to cell death at least in terms of LDH release as previously observed [34] and Annexin V/FVD eFluor780 staining; however, we could not exclude the involvement of cytopathic effects on the monolayer such as alteration of tight junction molecules among the cells [35].

The airway epithelial cells are a major source of chemokines involved in the host response to mycobacteria infection. It has been shown that recruitment of PMN by A549 alveolar epithelial cells is driven by the release of IL-8 upon their infection with laboratory strains, clinical isolates of *Mtb* [36], and *M. bovis* BCG [37]. Herein, we show that M strain induced low IL-8 secretion by bronchial cells and, as a consequence, its conditioned medium failed to attract PMN. In addition, pulmonary epithelial cells are also able to produce cytokines to shape a proper immune response. In this line, it has been demonstrated that uninfected Calu-6 constitutively produce high levels of TGF-*β* [38], and we observed that the infection with *Mtb* strains did not alter its production, as TGF-*β* is a potent anti-inflammatory mediator and its presence would be necessary to avoid an exacerbated innate immune response in the airway space [39]. In spite of this anti-inflammatory scenario, the secretion of TNF-*α* by Calu-6 cells was indeed increased by the infection with *Mtb* strains that was dependent on the ingested bacilli. In fact, M strain which has a lower infective ability than H37Rv-enhanced TNF-*α* secretion only at a MOI of 50. Considering that TNF-*α* is involved in PMN activation and priming of responses to secondary stimuli [40], we would speculate that the infection of bronchial cells with *Mtb* could modulate PMN effector responses to a second stimulus. In fact, our results show that CM from uninfected or *Mtb*-infected Calu-6 did not modify the expression of CD11b, an activation/degranulation marker [24] in resting PMN. Instead, the activation of PMN upon i-H37Rv stimulation was altered and it was dependent on the sources of CM, while H37Rv-CM increased CD11b expression, M-CM did not, and it was dependent on the content of TNF-*α* of each CM. As CD11b is translocated to the cell surface membrane during granule exocytosis as a consequence of PMN activation [24], our results suggest that soluble mediators from the *Mtb*-infected bronchial cells could potentiate PMN activation to a second stimuli via a differential TNF-*α* release.

It has been reported that TNF-*α* and GM-CSF do not directly induce the PMN oxidative burst but enhance ROS production triggered by fMLP [41]. In agreement, none of the CM employed directly activated the PMN oxidative burst, but they upregulated ROS production in a different manner in response to a second stimuli. Our results demonstrate that the higher TNF-*α* in the CM used as PMN primer, the stronger ROS production by PMN in response to PMA or i-H37Rv. Furthermore, our assays carried out that TNF-*α*-depleted or TNF-*α*-supplemented CM provide evidences on the impact of TNF-*α* release by bronchial epithelial cells on ROS production by PMN. Although it is controversial whether PMN can kill extracellular and intracellular *Mtb* bacilli, it is well established that these cells interact with and internalize mycobacteria [28, 42], being the predominant cell types infected with *Mtb* in lung samples from patients with TB [43]. In agreement with these studies, we observed that PMN internalized H37Rv bacilli despite the preincubation with CM suggesting that bronchial environment did not alter PMN ability to recognize and phagocytose *Mtb*. However, we observed that while control-CM and H37Rv-CM did not modify bacterial growth, M-CM enhanced H37Rv intracellular growth in PMN. Increased H37Rv replication in PMN pretreated with M-CM could be partially attributed to their low ability to produce intracellular ROS, despite its controversial role as microbicide [28]. Besides, as PMN exposed to the CM showed a lower NO production than the nontreated ones upon infection, we could speculate that in our system, reactive nitrogen intermediates would not be involved in H37Rv replication control, despite their bacteriostatic effect on *Mycobacterium tuberculosis* in vitro [29]. However, we cannot rule out that other mechanisms such as fusion of granules containing antimicrobial effectors with the *Mtb* phagosome could be altered in H37Rv-infected PMN exposed to M-CM. In this regard, M strain would manipulate in a bystander manner the PMN functionality by inhibiting TNF-*α* release from bronchial epithelial cells.

We observed that CM did not induce PMN death but the infection of PMN with H37Rv resulted in an increased cell death, and furthermore, only the pretreatment of PMN with control-CM, which showed the high levels of GM-CSF, delays PMN death. These results stay in line with those demonstrating that PMN quickly succumb to cell death upon H37Rv infection [28] and with that showing that GM-CSF released by primary bronchial epithelial cells increases the in vitro survival of human PMN. [44]. Finally, and in consonance with another study [45], the infection of PMN with H37Rv increases IL-8 amounts. However, pretreatment of PMN with H37Rv-CM or control-CM did not modify the IL-8 release upon infection with H37Rv. Interestingly, PMN preincubated with M-CM secreted higher IL-8 than that with the noninfected ones, which could be influencing the PMN recruitment.

In summary, we observed that although MDR *Mtb* strain M has a low rate of infection, it is able to survive within bronchial epithelial cells establishing an anti-inflammatory environment. Besides, M strain is a low inducer of IL-8 in bronchial epithelium minimizing PMN chemotaxis as well as an inhibitor of TNF-*α* secretion influencing on CD11b expression and ROS production by PMN. Furthermore, CM from M-infected bronchial cells enhance intracellular mycobacterial replication within PMN. Upon death of *Mtb*-infected PMN, a high number of mycobacteria could be released to the lung compartment infected by M strain. On the other hand, the fact that M-infected bronchial epithelium enhances IL-8 release by *Mtb*-infected PMN would result in a sustained recruitment of PMN and other immune cells to the site of infection which could be a double-edged sword for the host.

## 5. Conclusion

The adhesion/invasion of *Mtb* to the bronchial epithelium depends on the infecting bacterial genotype which will induce a differential immune response given by soluble mediators released by the epithelium, altering in turn the functionality of PMN in a bystander manner.

## Supplementary Material

Supplementary Figure 1: Calu-6 cells monolayers were infected or not with H37Rv or M strains at a MOI of 5 for 2h and then cultured for further 18h. Detached cells were stained with specific antibodies (open histograms, non-infected: black, M‐infected: blue and H37RV‐infected cells: red) or with isotype‐matched antibodies (grey full histograms) and tested for their expression of CD11b, TLR2, Mannose receptor (MR) and CD54 by FACS. Histogram plots from a representative experiment of 8 done are shown. Supplementary Figure 2: Conditioned medium (CM) from 18h cultured uninfected (陹) or H37Rv‐ (H37Rv‐CM 陹) or M‐infected (M‐CM 防) Calu‐6 cells at a MOI 5‐50 were tested for A. TNF‐*α*, GM‐CSF and IL‐8 by ELISA. Results are expressed as pg/ml (mean ŷ SEM).
